# Response to lower dose TNF inhibitors in axial spondyloarthritis; a real-world multicentre observational study

**DOI:** 10.1093/rap/rkaa015

**Published:** 2020-05-13

**Authors:** Liz Van Rossen, Antoni Chan, Annie Gilbert, Karl Gaffney, Claire Harris, Pedro M Machado, Liliana R Santos, Raj Sengupta, Paul Basset, Andrew Keat

**Affiliations:** r1 Department of Research and Development, East Kent Hospital University Foundation Trust, Canterbury; r2 Department of Rheumatology, Royal Berkshire Hospital, Reading; r3 AK Gilbert Ltd, Brighton; r4 Department of Rheumatology, Norfolk and Norwich University Foundation Trust, Norwich; r5 Department of Rheumatology, Northwick Park Hospital, London North West University Healthcare NHS Trust, London; r6 Department of Rheumatology & Queen Square Centre for Neuromuscular Diseases, University College London Hospitals NHS Foundation Trust, London; r7 Department of Rheumatology, Royal United Hospitals Trust, Bath; r8 Statsconsultancy Ltd, Amersham, UK

**Keywords:** axial spondyloarthritis, biologic therapies, dose reduction, TNF inhibitor treatment, outcome measures

## Abstract

**Objective:**

Dose optimization of TNF inhibitors in axial spondyloarthritis (axSpA) is attractive, but it is unclear for which patients this approach might be appropriate.

**Methods:**

Seventy-one patients with axSpA, from six UK centres, were identified who had reduced their dose of TNF inhibitor after being considered to be stable responders. All completed a questionnaire concerning their approach to and experience of dose reduction. Data on patient characteristics, metrology and CRP were retrieved retrospectively from patient records.

**Results:**

Over 2 years of observation, 60 (84.5%) remained (REM) on reduced-dose medication and 11 (15.5%) reverted (REV) to the original dose. The overall mean dose reduction was 39% for REM patients and 44% for REV patients. Both groups initially responded in a similar manner to treatment, but the data showed a trend that younger women were more likely to revert. Neither BMI nor smoking was associated with continued low-dose responsiveness. Eight of the 11 REV patients reverted by 6 months. None reached criteria of secondary drug failure, and all regained control after increasing back to the original dose. Most patients in both groups reached the decision to reduce the dose jointly with clinicians. A preference for taking the reduced dose was not associated with low-dose drug survival.

**Conclusion:**

Many patients with axSpA remain well symptomatically after stepping down the dose of TNF inhibitor, but young women are less likely to do well on a reduced dose. Dose reduction should be one element of the management of patients with axSpA.

**Key messages**

- Of stable responders to TNF inhibitor, 84.5% remained on a mean 39% reduced-dose medication over 2 years of observation.
- Younger axial spondyloarthritis patients and females were associated with risk of reversion to full-dose treatment.
- Dose reduction in axial spondyloarthritis should be part of dose-optimization strategies.

## Introduction

Several studies have shown that withdrawal of TNF inhibitor (TNFi) after a good response in patients with axial spondyloarthritis (axSpA) results in relapse in the majority of patients [1–3]. However, clinical experience indicates that some patients with axSpA respond initially to lower-dose treatment [4–6] and that some of those who respond well to full-dose TNFi treatment are able to reduce the dose and remain symptomatically well, with or without concurrent MTX [7–10]. Dose optimization is attractive on cost and safety grounds, but a number of important unresolved questions prevent its recommendation; chief of these is whether the potential benefits of TNFi treatment, besides symptom control (prevention of long-term structural damage and long-term functional impairment), can be maintained at lower doses. Of equal importance is the identification of patients who are likely to do well on reduced-dose treatment, or even treatment withdrawal, and those in whom such an approach has a high likelihood of failure. Dose reduction carries the risk of disease flare; this may reflect incidental secondary failure of TNFi treatment [11, 12], but experience of restarting full-dose treatment after treatment dose reduction suggests that restoration of response is usual. 

In the absence of clear data to support such dose-reduction strategies, dose reduction has occurred largely on an *ad hoc* basis. Some patients have simply chosen to use the minimum dose because of personal preference, whereas some rheumatology units in the UK have allowed dose reduction where patients have expressed a preference. These changes need to be seen in the context of limited compliance with TNFi dosing reported in RA and psoriasis, ranging from 40% to >80% [13, 14]. Such *ad hoc* changes allow only limited conclusions to be drawn about the pros and cons of dose reduction, but they might help to identify individuals in whom such strategies might be successful. Complex therapeutic trials might eventually help to resolve these issues, whereas real-world observational studies can help to provide some guidance to clinicians and patients who need it now.

At six rheumatology units within the UK, patients with axSpA who were noted to have reduced the dose of TNFi medication after a good response were investigated. The aim of the study was to explore patient- and disease-associated factors predictive of long-term success and failure of reducing the doses of TNFi medication over a 2-year period.

## Methods

Seventy-one patients, from six UK centres, with a diagnosis of axSpA and fulfilling classification criteria for AS (modified New York criteria [15]) or axSpA (Assessment of SpondyloArthritis international Society [ASAS] criteria [16]) and who had reduced their dose of TNFi, were identified. All were considered to be stable responders to TNFi; their responses fulfilled National Institute for Health and Care Excellence criteria and were maintained for ≥6 months by the time of dose reduction. Each was observed for 2 years after dose reduction, with the outcome of interest being the time to reversion to the standard dose**.** No planned dose-reduction regimen was used; patients either decided for themselves or were advised on an *ad hoc* basis by their treating clinicians.

After obtaining ethical approval, all patients, whether now on reduced-dose treatment or having reverted to full-dose treatment, were asked to complete a questionnaire. This asked about ethnicity, the way in which dose-reduction decisions were taken, including the input of health-care professionals, the confidence with which patients took or accepted the decision, perceived effects of dose reduction on symptoms, lifestyle, work and sleep, effects on any associated conditions and any changes in concurrent medication. All patients were also asked to quantify cigarette smoking, past and present.

Data on age, height, weight and BMI, disease duration, duration and doses of TNFi treatment and responses as measured by the BASDAI, BASFI and BASMI over time were collected from departmental clinical records. Each patient was assigned a random number, meaning that, after linkage of the two data sets, analysis was carried out on anonymized data.

Thirty-seven patients were treated with adalimumab, 20 with etanercept, seven with infliximab and seven with golimumab. All patients started treatment on standard recommended doses and frequency of administration. All patients were taking originator drugs at the time of the study; none was receiving a biosimilar. The proportion of dose reduction was calculated as a percentage, in milligrams per month, of the original, standard dose. For patients receiving infliximab, the percentage dose reduction was calculated from the milligrams per kilogram administered and the interval between infusions. All patients completed BASDAI and BASFI questionnaires at each visit, with annual BASMI measurement. CRP levels were measured frequently. Individuals who continued to take reduced-dose treatment throughout the observation period were designated remainers (REM) and those who reverted to full-dose treatment during it were designated reverters (REV). For REV patients, the duration of reduced-dose treatment was recorded. The mean dose reduction for each of the four agents was 39% for adalimumab, 39% for etanercept, 26% for golimumab and 46% for infliximab. The overall mean dose reduction was 39% for REM patients and 44% for REV patients.

Data were collected at six time points: (1) immediately before starting TNFi therapy; (2) at the point of dose reduction; (3) at the point of reversion to full-dose treatment (REV only); (4) 6 months after dose reduction; (5) 12 months after dose reduction; and (6) 24 months after dose reduction. Time point 3 was notional, because reversion could occur at any point over the observation period.

The analysis of the time to reverting to the standard dose was performed using survival analysis methods. The length of follow-up was recorded only up to 24 months, meaning that only patients reverting within the first 24 months were considered; those who reverted after 24 months were considered not to have reverted. Kaplan–Meier methods were used to plot the proportion remaining on the low-dose over time and to quantify the proportion at key time points in the follow-up period. Patient factors associated with the time to reversion were analysed using Cox regression.

## Results

Of the 71 participants, 56 (78.9%) were male, aged 28–71 (mean 51.1) years, and 15 (21.1%) female, aged 24–71 (mean 49.9) years. Sixty-one were white, eight were of South Asian origin and two were of other ethnicity. Within the 24-month period, 60 (84.5%) patients were REM and 11 (15.5%) were REV. The mean disease duration in these two subgroups was 19.4 and 22.2 years, respectively. REM patients had taken TNFi for a mean of 3.6 years compared with 1.8 years for REV patients before dose reduction. Nine (16.1%) patients had diagnosed IBD, 11 (19.6%) had psoriasis and 11 (19.6%) had previously had episodes of uveitis. Eight (11.3%) were current smokers and 38 (53.5%) others had smoked at some time (ever smokers); 25 (35.2%) had never smoked. Fifty-four (76.1%) patients were employed or self-employed, 3 (4.2%) were work-disabled and 13 (18.3%) were retired.

Demographics, including work and associated co-morbidities of the whole group, REM and REV patients are demonstrated in Table 1. It can be seen that REM and REV patients have broadly similar demographics. Further analysis of age, sex, baseline BASDAI and CRP are presented in Table 2.

**Table 1:** Patient characteristics

Characteristic	Total (*n* = 71)	**REM** **(*n* = 60)**	REV (*n* = 11)
Female, *n* (%)	15 (21.1)	10 (16.6)	5 (45.5)
Age, mean (range), years	50.5 (24–72)	51.2 (26–71)	46.8 (24–72)
BMI, mean (range), kg/m^2^	26.8 (19–37)	26.6 (19–37)	27.6 (24–35)
Duration of disease, mean (range), years	19.9 (6–41)	19.4 (6–41)	22.2 (15–31)
Duration of TNFi at dose reduction, mean (range), years	3.9 (0.3–10.9)	3.6 (0.3–10.9)	1.8 (1.9–9.3)
Baseline BASDAI	–	6.2	5.7
Baseline CRP	–	14.5	9.4
Ethnicity, *n* (%)			
Caucasian	61 (85.9)	52 (86.7)	9 (81.8)
South Asian	8 (11.3)	6 (10)	2 (18.2)
Other	2 (2.8)	2 (3)	0 (0)
Co-morbidities, *n* (%)			
Uveitis	11 (19.6)	9 (15)	2 (18.2)
Psoriasis	11 (19.6)	8 (13.3)	3 (27.3)
IBD	9 (16.1)	9 (15)	0 (0)
Work status^a^, *n* (%)			
Employed	35 (50)	28 (46.7)	7 (63.6)
Self-employed	19 (27.1)	17 (28.3)	2 (18.2)
Not working	16 (22.9)	14 (23.3)	2 (18.2)
Smoking status, *n* (%)			
Current smoker	8 (11.3)	8 (13.3)	0 (0)
Ever smoker	38 (53.5)	33 (55)	5 (45.5)
Never smoker	25 (35.2)	19 (31.7)	6 (44.5)

a Data missing from one patient. REM: remainers; REV: reverters.

**Table 2:** Regression analyses examining factors associated with time to revert to standard dose

Variable	Category/term	Hazard ratio (95% CI)	*P*-value
Age^a^	Linear term	0.94 (0.58, 1.48)	0.05
	Squared term	1.38 (1.00, 1.91)	
Sex	Male	1	0.04
	Female	3.44 (1.05, 11.3)	
BASDAI (baseline)	–	0.72 (0.43, 1.19)	0.20
CRP (baseline)	–	1.02 (0.79, 1.31)	0.89

a Hazard ratio is given for a 10-year increase in age.

**Fig. 1:**
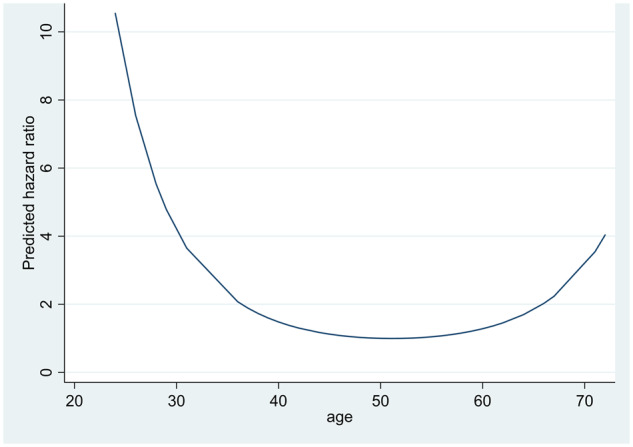
Risk of reverting to original dose by age The results also suggest a significant association between sex and the time to reversion. Females were found to have a significantly increased risk of reverting than males. The hazard (or risk) of reverting at any time was more than three times higher in females than males. Statistical analysis provides no evidence that either the BASDAI or CRP level was associated with the time to revert to the original dose. Two remain (REM) patients reported worsening of co-morbidity symptoms (one IBD, one psoriasis), but this did not lead to reversion. No revert (REV) patients reported worsening of co-morbidity symptoms. The proportion of patients remaining on the original dose over the study period is shown in the Kaplan–Meier plot in Supplementary Fig. S1, available at *Rheumatology Advances in Practice* online.

Age was also associated with reversion (*P* = 0.05), but the data suggested that there was not a consistent trend with increasing age. Thus, to provide the best fit for the data, it was necessary to include both linear and squared terms for age, with the results being best interpreted graphically. Fig. 1 shows how the hazard ratio for age changes with time. The risk of reverting to the low dose is presented relative to a person of average age (50 years). The graph suggests that the highest risk of reverting was for the youngest subjects. The risk decreased with increasing age up to ∼50 years. For subjects aged >60 years, there was an increased risk of reverting with further increase in age. The results also suggest a significant association between sex and the time to reverting. Females were found to have a significantly increased risk of reverting than males. The hazard (or risk) of reverting at any time was more than three times higher in females than males.

**Fig. 2:**
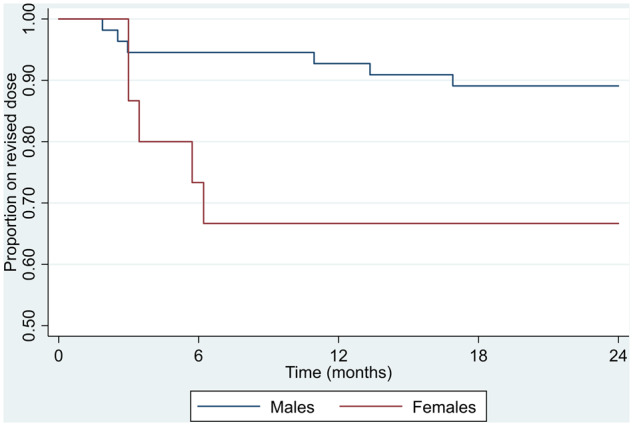
Kaplan–Meier plot of time to reversion to the original dose by sex Neither baseline BASDAI score nor CRP level at the initiation of TNFi treatment was associated with reversion. It can be seen that both groups responded equally to treatment and exhibited similar scores in all measures at dose reduction and 6 months.

Graphical illustrations of the results for the total number of patients are shown in Supplementary Fig. S1, available at *Rheumatology Advances in Practice* online, and in Fig. 2. Statistical analysis showed no evidence that either the BASDAI or CRP was associated with the time to revert to the original dose.

Two REM patients reported worsening of co-morbidity symptoms (one IBD, one psoriasis), but this did not lead to reversion. No REV patients reported worsening of co-morbidity symptoms.

The proportion of patients remaining on the original dose over the study period is shown in the Kaplan–Meier plot (Supplementary Fig. S1, available at *Rheumatology Advances in Practice* online). It can be seen that 8 of the 11 patients reverted by 6 months and that 84% of patients remained on the low dose at 24 months.

Analyses of factors associated with time to revert to standard dose indicated that females had a significantly increased risk of reverting compared with males, with the risk of reverting at any time being more than three times higher (*P* = 0.04). This is illustrated in Fig. 2.

**Fig. 3:**
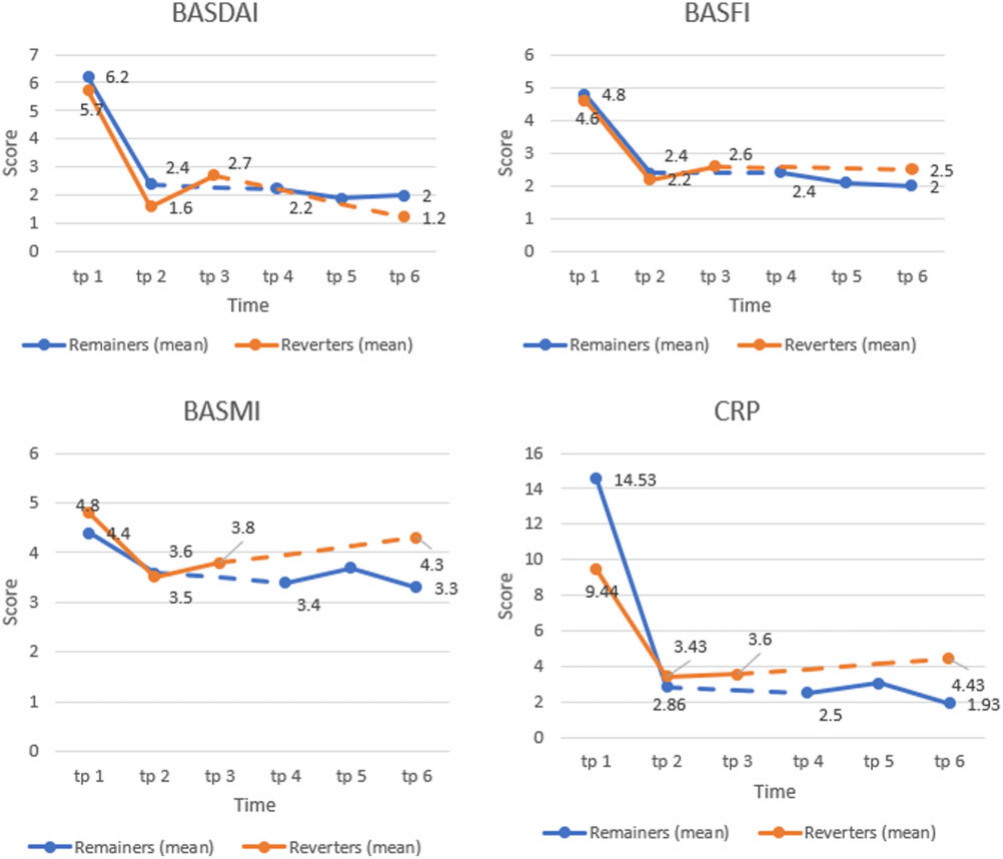
BASDAI, BASFI, BASMI and CRP levels from the time of dose reduction (time point 1) to 24 months (time point 2) It can also be seen that scores for remain (REM) patients were stable or decreased over the observation period from dose reduction, with mean BASDAI scores reduced from 2.4 to 2.0 (20.8%), mean BASFI scores reduced from 2.4 to 1.9 (16.6%), mean BASMI scores reduced from 3.5 to 3.3 (5.7%) and mean CRP scores decreased from 3.4 to 1.9** **mg/dl (87.2%). The mean BASDAI scores of reversion (REV) patients from dose reduction to dose reversion decreased from 1.6 to 1.2 (25%), but over this period the mean BASFI scores increased from 2.2 to 2.5 (13.6%), mean BASMI scores increased from 3.5 to 4.3 (22.8%) and mean CRP scores also increased modestly from 3.4 to 4.4** **mg/dl (29.4%).

Neither the baseline BASDAI score nor the CRP level at the initiation of TNFi treatment was associated with reversion. It can be seen in Fig. 3 that both groups responded equally to treatment and exhibited similar scores in all measures at dose reduction and 6 months. Fig. 3 also shows that scores for REM patients then remained stable or decreased over the observation period from dose reduction, with mean BASDAI scores reduced from 2.4 to 2.0 (20.8%), mean BASFI scores reduced from 2.4 to 1.9 (16.6%), mean BASMI scores reduced from 3.5 to 3.3 (5.7%) and mean CRP scores decreased from 3.4 to 1.9 mg/dl (87.2%). The mean BASDAI scores of REV patients from dose reduction to dose reversion decreased from 1.6 to 1.2 (25%), but over this period the mean BASFI scores increased from 2.2 to 2.5 (13.6%), mean BASMI scores increased from 3.5 to 4.3 (22.8%) and mean CRP scores also increased modestly from 3.4 to 4.4 mg/dl (29.4%).

### Effect of dose reduction on work, convenience, sleep and concomitant medication

It can be seen in Table 3 that most patients in both groups reached the decision jointly with clinicians. Fifty-one (85%) REM patients either preferred or were indifferent about the decision to reduce the dose, but six (10%) REM and seven (63.6%) REV patients did not prefer reducing the dose. Preference for taking the reduced dose was associated with success of the low-dose regimen. REV patients were more likely to increase pain medication, including NSAIDs.

**Table 3:** Patient aspects of dose reduction

Variable	Category	**REM (*n* = 60)**	REV (*n* = 11)
Decision to reduce dose, *n* (%)	Jointly with clinician	40 (66.6)	8 (72.7)
	Patient decision	11 (18.3)	1 (9.1)
	Health professional	7 (11.7)	1 (9.1)
Preference for lower dose, *n* (%)	Preferred	36 (60.0)	2 (18.2)
	No difference	15 (25.0)	1 (9.1)
	Did not prefer	6 (10.0)	7 (63.6)
Undisturbed sleep, h	–	5.9	6.1
Dose of pain medication, *n* (%)	Less	6 (10.0)	1 (9.1)
Since reduction, *n* (%)	No change	41 (68.3)	1 (9.1)
	More	9 (15.0)	8 (72.7)

REM: remainers; REV: reverters.

## Discussion

Despite the small numbers, these findings suggest signals worthy of further data collection and analysis. Principally, the study suggests that, amongst these selected patients with axSpA, 84% continued to respond well to TNFi treatment over 24 months in spite of a mean dose reduction of 38%, but that age and sex are associated with drug survival of reduced-dose TNFi regimens. There are grounds for cautiously predicting that older male patients with raised pre-treatment levels of CRP who have achieved the National Institute for Health and Care Excellence response to TNFi treatment for 6 months are likely to remain symptomatically controlled after dose reduction of ∼39%. It might be that reducing the dose after a longer period of stable response, perhaps 12 months, would greatly reduce the risk of reversion. It appears likely that patients who are confident with the reduced-dose approach, whose decision has been shared with the clinical team and for whom the opportunity to revert is readily available are more likely to remain well on reduced-dose treatment.

The patients in the present study were typical of UK hospital axSpA populations, although the low prevalence of women might reflect the lower proportion of patients with non-radiographic axSpA receiving TNFi treatment at the time of recruitment.

Notably, at the point of reversion, REV patients did not reach BASDAI levels indicative of secondary drug failure, and all of the REV patients regained control after dose reversion. This was preceded by modest increases in BASDAI, BASFI, BASMI and CRP levels; the worsening of symptoms but maintenance of low CRP levels raises questions about the mechanisms underpinning dose reversion. Lower CRP levels have been reported in women with axSpA [17], but the explanation is unclear. Unsurprisingly, REV patients took more analgesic medication than REM patients after dose reduction, commensurate with worse symptoms.

In this small sample, 8 of the 11 REV patients reverted within 6 months. REV patients had taken biologic treatment for less time than REM patients, suggesting that dose reversion is more likely early in TNFi treatment and soon after dose reduction. It might be significant that all responded symptomatically to increasing the dose. No clear influence of smoking has been shown, with approximately half the patients in each group being ever smokers and only a few being current smokers. The lack of effect of smoking might be attributable to the small sample size, but it is consistent with the findings that smoking does not influence response to TNFi in axSpA [18].

The patient’s view of dose reduction is clearly relevant to the success of dose optimization; although this study is small, some important issues have been identified. Most patients in both groups made the dose-reduction decision jointly with clinicians. More REV patients than REM patients, however, expressed concern about the dose reduction, suggesting that being ill at ease with dose reduction might have influenced survival of low-dose treatment in this group. Holmes *et al.* [19] reported that most patients are interested in reducing the dose of TNFi but that fear of flare and inability to access expert advice tempered this enthusiasm. Hewlett *et al.* [20] also found that patients with axSpA express anxiety about dose reduction but that clear rationale, shared decision-making and control over the dose they take improves confidence. Our findings would support these views.

In the present study, the process of shared decision-making was not structured or formalized, and the decision to revert the dose was deliberately based on patient choice. In further studies, it would be helpful to draw up clear criteria for both dose reduction and dose reversion. It would also be crucial to record the nature of symptoms leading to dose reversion precisely, in order that typical spondylitic symptoms could be separated from fibromyalgic features, peripheral joint symptoms and other symptoms that might be unrelated to the primary disease. Likewise, it would be useful to categorize patients before dose reduction with regard to such features as anxiety that might influence readiness to dose revert and to agree to set the decision in the context of objective measures, including BASDAI and CRP. This might help to establish whether the desire to dose revert reflected disease activity or other factors. Ultimately, patient choice might still be a sound basis for dose changes in patients with axSpA receiving biologic therapies. However, it is crucial that the patient is informed in a precise and standardized way, that the symptoms and measures of disease activity are precisely recorded at the time of decisions to change the dose and that there is a justified relationship of trust between patient and condition.

In the treatment of axSpA, the treat-to-target approach [21, 22], with its uncertain targets but implication of careful choice of first drug, dose escalation and drug switching, is appealing but only part of the problem of optimized treatment [23]. Keeping the target in mind but maintaining it with minimal treatment is an integral part of the approach. In RA, dose-reduction strategies are frequently successful [24] and advocated in its treatment [25, 26], but this is not currently the case with axSpA [27].

In axSpA, therefore, it is important to define and understand both flare and relapse; both may reflect changes in symptoms caused by either increased inflammatory activity or non-inflammatory mechanisms. It is of interest that none of the REV patients reached criteria for secondary drug failure. We agree with Edwards *et al.* [28] that there is also a need to define the targets of not only induction-phase full-dose remission (or low disease activity) but also maintenance-phase remission low disease activity in patients with axSpA.

Expenditure on medication and drug delivery was not assessed in the present study, and absolute figures would now be influenced heavily by the introduction of biosimilars. However, it is worthy of comment that of the 60 REM patients, the mean dose reduction was 39%, offering a substantial saving on medication costs. This is clearly important in countries where drug costs are borne by health services or insurers but might be critically so in those where drug costs, hence access to treatment, are borne by the patients themselves.

In this study, any individuals who explicitly did not wish to take reduced-dose treatment were excluded. Clearly, extended follow-up will be necessary, and the likelihood of regaining low disease activity after dose reversion needs to be clarified; however, in this study, all REV patients regained symptom control and none switched to an alternative biologic agent. It will be pertinent to consider further step-down approaches and to develop further predictors of their success for both TNFi and other biologic agents.

## Supplementary Material

rkaa015_Supplementary_Data
