# Supplementary material for: Response to lower dose TNF inhibitors in axial spondyloarthritis; a real-world multicentre observational study
Source: Rheumatol Adv Pract. 2020 May 13;4(2):rkaa015. doi: 10.1093/rap/rkaa015 (PMC7415263; doi:10.1093/rap/rkaa015)
Supplement: rkaa015_Supplementary_Data [file rkaa015_supplementary_data.zip › RAP-20-021.R1_Supplementary_Figure document.docx]

**SUPPLEMENTARY MATERIAL**

**Supplementary figure legend**

**Supplementary Figure S1: Kaplan-Meier plot of time to reverting to original dose (reduced y-axis)** It can be seen that 8 of the 11 patients reverted by 6 months and that 84% of patients remained on the low dose at 24-months.

Analyses of factors associated with time to revert to standard dose indicate that

females were found to have a significantly increased risk of reverting than males, the risk of reverting at any time being over 3 times higher (p=0.04). This is illustrated in figure 2.
