# Supplementary figures and images for: Response to lower dose TNF inhibitors in axial spondyloarthritis; a real-world multicentre observational study
Source: Rheumatol Adv Pract. 2020 May 13;4(2):rkaa015. doi: 10.1093/rap/rkaa015 (PMC7415263; doi:10.1093/rap/rkaa015)

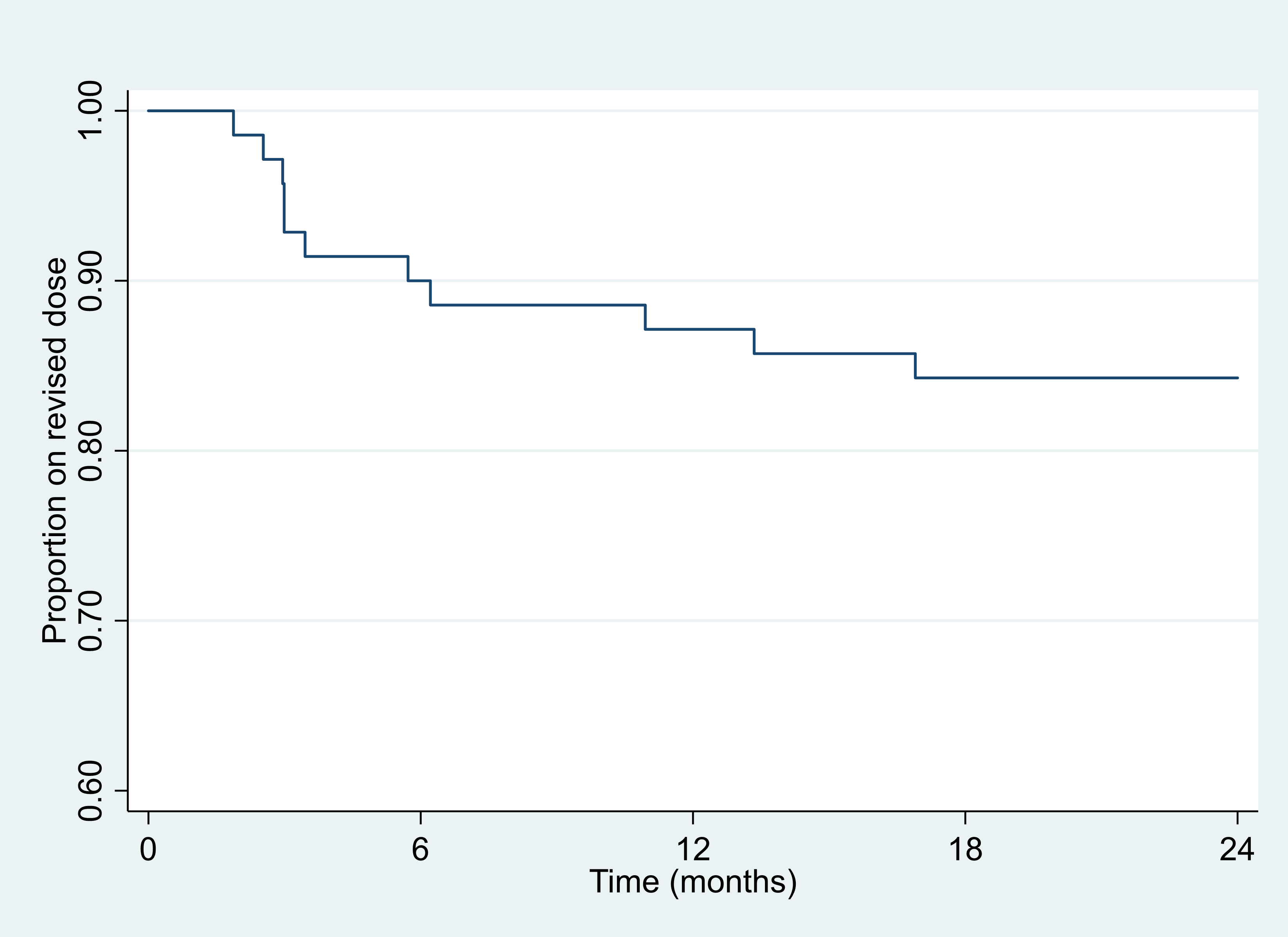

Supplement: rkaa015_Supplementary_Data [file rkaa015_supplementary_data.zip › Sup Fig Rheumap FINAL.png]
